# O-GlcNAcylation regulates ischemia-induced neuronal apoptosis through AKT signaling

**DOI:** 10.1038/srep14500

**Published:** 2015-09-28

**Authors:** Jianhua Shi, Jin-hua Gu, Chun-ling Dai, Jianlan Gu, Xiaoxia Jin, Jianming Sun, Khalid Iqbal, Fei Liu, Cheng-Xin Gong

**Affiliations:** 1 Jiangsu Key Laboratory of Neuroregeneration, Co-Innovation Center of Neuroregeneration, Nantong University, Nantong, Jiangsu 226001, China; 2 Department of Biochemistry, Nantong University Medical School, Nantong, Jiangsu 226001, China; 3 Department of Neurochemistry, Inge Grundke-Iqbal Research Floor, New York State Institute for Basic Research in Developmental Disabilities, Staten Island, New York 10314, United States of America; 4 Department of Pathophysiology, Nantong University Medical School, Nantong, Jiangsu 226001, China

## Abstract

Apoptosis plays an important role in neural development and neurological disorders. In this study, we found that O-GlcNAcylation, a unique protein posttranslational modification with O-linked β-N-acetylglucosamine (GlcNAc), promoted apoptosis through attenuating phosphorylation/activation of AKT and Bad. By using co-immunoprecipitation and mutagenesis techniques, we identified O-GlcNAc modification at both Thr308 and Ser473 of AKT. O-GlcNAcylation-induced apoptosis was attenuated by over-expression of AKT. We also found a dynamic elevation of protein O-GlcNAcylation during the first four hours of cerebral ischemia, followed by continuous decline after middle cerebral artery occlusion (MCAO) in the mouse brain. The elevation of O-GlcNAcylation coincided with activation of cell apoptosis. Finally, we found a negative correlation between AKT phosphorylation and O-GlcNAcylation in ischemic brain tissue. These results indicate that cerebral ischemia induces a rapid increase of O-GlcNAcylation that promotes apoptosis through down-regulation of AKT activity. These findings provide a novel mechanism through which O-GlcNAcylation regulates ischemia-induced neuronal apoptosis through AKT signaling.

Cerebral ischemia is a condition that occurs when an insufficient blood flow to the brain does not meet its metabolic demand, which leads to poor oxygen supply and often to cerebral infarction or ischemic stroke. Many studies have shown that apoptosis plays an important role in ischemia-induced neuronal death and the loss of neural function in the affected area^1^. However, how cerebral ischemia causes apoptosis remains elusive.

Protein O-GlcNAcylation is a unique type of glycosylation of nucleocytoplasmic proteins and refers to the enzymatic transfer of β-N-acetylglucosamine (GlcNAc) from the UDP-GlcNAc donor to the hydroxyl group of serine/threonine residues of proteins via an O-glycosidic bond^2^. This process is catalyzed by O-linked N-acetylglucosamine transferase (O-GlcNAc transferase, OGT), and the O-GlcNAc group on proteins can be removed with the catalysis of β-N-acetylglucosaminidase (O-GlcNAcase, OGA). Protein O-GlcNAcylation is regulated dynamically by these two enzymes and the intracellular concentration of UDP-GlcNAc, a product of glucose metabolism through the hexosamine biosynthetic pathway^3^. Protein O-GlcNAcylation is ubiquitous in living systems and appears to regulate numerous cellular signaling and functions, including transcription, translation, protein degradation, cell signaling, cell trafficking, cell cycle control, and apoptosis^2^,^3^,^4^.

Protein O-GlcNAcylation is known to be a sensor of intracellular glucose metabolism and cellular stress^5^. It is reasonable to speculate a marked alteration of protein O-GlcNAcylation under ischemia as the result of insufficient blood flow and ischemia-induced cellular stress. Because O-GlcNAcylation may regulate numerous cellular functions, we hypothesize that altered O-GlcNAcylation resulting from ischemia regulates neuronal apoptosis and degeneration. Our hypothesis is supported by previous findings of a regulatory role of O-GlcNAcylation in apoptosis in pancreatic β-cells^6^,^7^ and in myocardial ischemic injury^8^,^9^,^10^. Whether neuronal apoptosis and cerebral ischemia are regulated by O-GlcNAcylation was not previously investigated.

In this study, we investigated the role of O-GlcNAcylation in ischemia-induced apoptosis and neuronal death in cultured cells and in a mouse model of middle cerebral artery occlusion (MCAO). We found that cerebral ischemia-induced neuronal cell death involved O-GlcNAcylation of AKT likely at Thr308 and Ser473 and phosphorylation/activation of AKT and Bad. These findings indicate that O-GlcNAcylation regulates ischemia-induced neuronal apoptosis through the AKT signaling pathway.

## Results

### Up-regulation of O-GlcNAcylation promotes cell apoptosis

**Figure 1:**
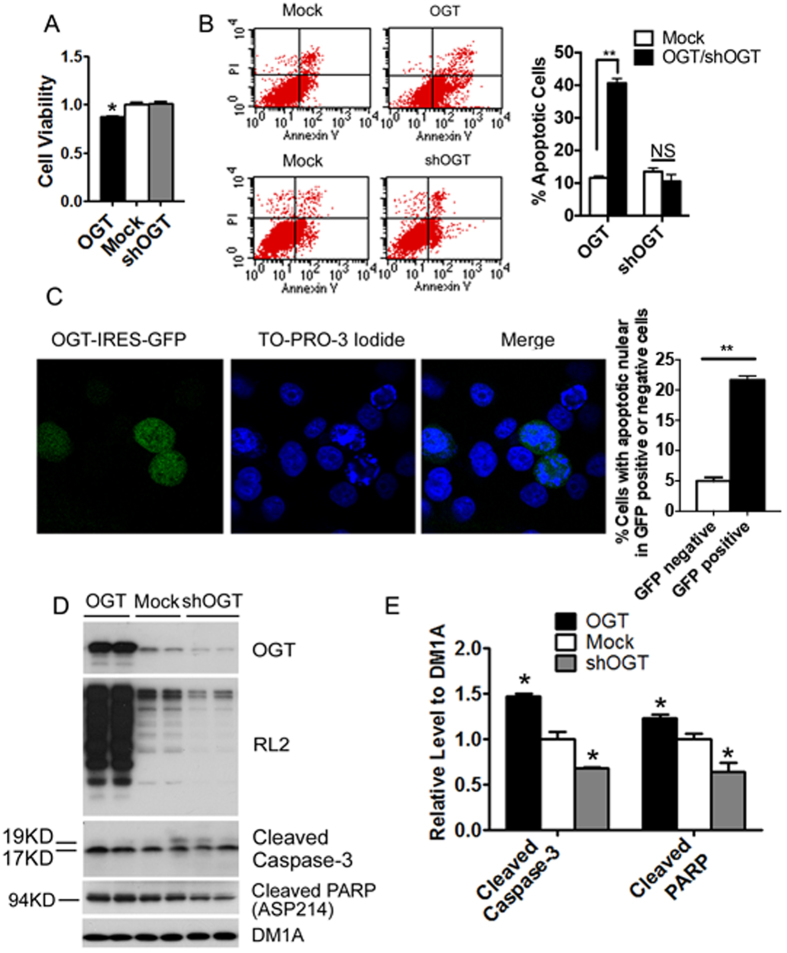
Up-regulation of O-GlcNAcylation promotes apoptosis. (**A**) HEK-293FT cells were transfected with OGT or shOGT for 48 h, and the cell viability was determined by CellTiter 96® Non-Radioactive Cell Proliferation Assay. (**B**) The OGT-, shOGT- or mock-transfected cells were stained with Annexin V and propidium iodide (PI) and then analyzed by using a flow cytometer. The percentage of apoptotic cells over the transfected cells is shown in the bar graph. (**C**) HeLa cells were transfected with pHRST-OGT-IRES-GFP for 48 h and then stained with TO-RPO-3 iodide. The ratios of the number of apoptotic nuclei over that of GFP-negative and -positive cells are shown in the bar graph. (**D**) Lysates of HEK-293FT cells after transfection with OGT or shOGT for 48 h were analyzed by Western blots developed with antibodies indicated at the right side of the blots. (**E**) Densitometric quantifications of the cleaved (activated) caspase 3 and PARP in the blots, as shown in panel D, after normalization with the tubulin levels (DM1A blot). Data are presented as mean ± SD (n = 6). *p < 0.05, **p < 0.01 vs. the mock group.

The effect of O-GlcNAcylation on apoptosis is controversial^8^,^11^,^12^,^13^,^14^. To learn whether O-GlcNAcylation plays an active role in cell apoptosis, we investigated cell viability and apoptosis after over-expression or knock-down of OGT. We observed that OGT over-expression in HEK-293FT cells for 48 h reduced cell viability significantly, but transfection with shOGT did not change the viability (Fig. 1A). Cytometric analyses indicated a two-fold increase in the apoptotic cells, as counted by the Annexin V-positive and PI-negative cells, 48 h after OGT transfection, but there was no significant change after transfection with shOGT (Fig. 1B). OGT over-expression also promoted apoptosis in HeLa cells, as evidenced by nuclear fragmentation and chromatin condensation in the OGT-IRES-GFP-transfected cells stained with TO-PRO-3 iodide nuclear staining (Fig. 1C). Quantitative assessment indicated that approximately 20% of the OGT-transfected cells became apoptotic under this condition, which was approximately 4 times higher than that in the mock-transfected cells (Fig. 1C, graph).

To confirm the role of O-GlcNAcylation in promoting apoptosis, we studied the activation/cleavage of caspase 3 and Poly-(ADP-ribose)-polymerase (PARP) in the lysates of HEK-293FT cells after transfection with OGT or shOGT for 48 h. We found that elevation of O-GlcNAcylation, as the result of OGT over-expression, increased the levels of both cleaved caspase 3 and cleaved PARP (Fig. 1D,E). In contrast, lowering O-GlcNAcylation, as the result of OGT knock-down, decreased the cleavage of these two apoptosis marker proteins. Taken together, these results indicate that up-regulation of protein O-GlcNAcylation promotes cell apoptosis.

### O-GlcNAcylation promotes apoptosis through attenuating phosphorylation/activation of AKT and Bad

**Figure 2:**
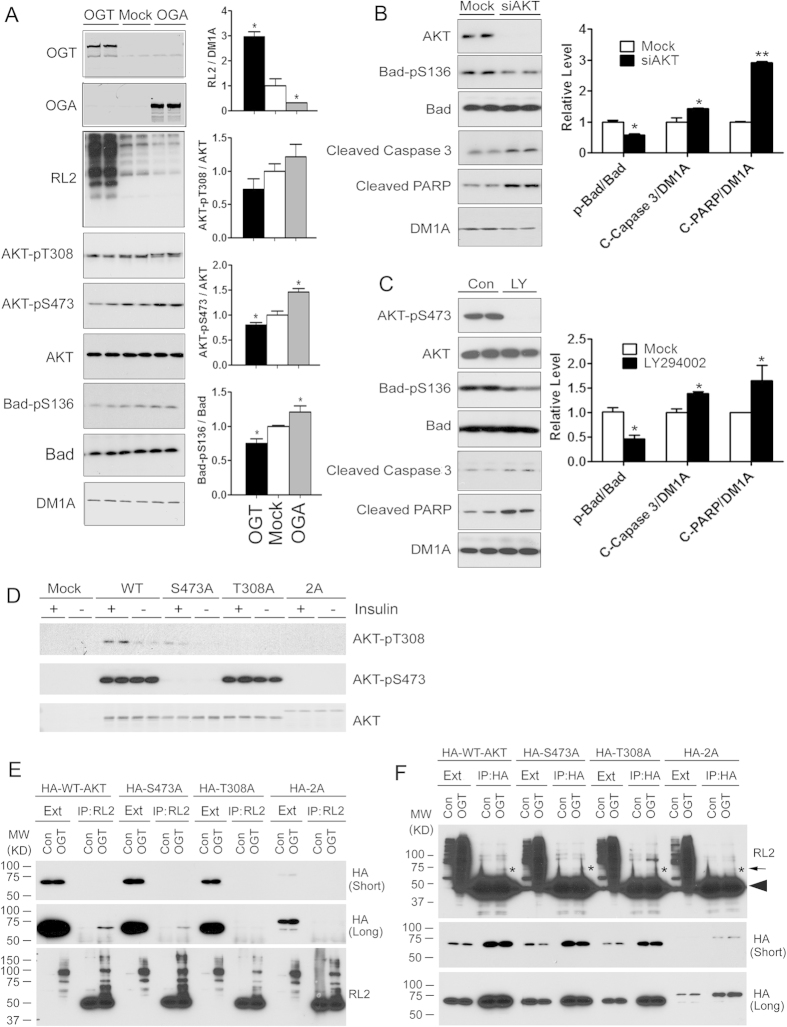
O-GlcNAcylation induces apoptosis through suppression of phosphorylation of AKT at Thr 308 and Ser473. (**A**) HEK-293FT cells were transfected with OGT or OGA for 48 h, and the cell lysates were subjected to Western blots developed with the antibodies indicated at the left side of the blots. Densitometrical quantifications of the blots are shown at the right side of the blots. (**B**,**C**) Western blots of the lysates of HEK-293FT cells after mock transfection or transfection with AKT siRNA for 48 h (**B**), or after treatment with AKT inhibitor LY294002 (20 μM) for 12 h (**C**). Densitometrical quantifications of the blots are shown at the right side of the blots. Data are presented as mean ± SD (n = 6). *p < 0.05 vs. control group. (**D**) HEK-293FT cells were transfected with HA-AKT, AKT_T308A_, AKT_S473A_, or AKT_2A_ for 36 h, followed by withdrawal of serum from the culture medium for another 12 h. Then, the cells were treated with 100 nM insulin for 30 min, and the cell lysates were analyzed by Western blots developed with antibodies indicated at the right side of the blots. (**E**) HEK-293FT cells were transfected with HA-WT-AKT and the indicated mutants for 48 h, followed by immunoprecipitation of O-GlcNAcylated proteins from the cell extracts using monoclonal antibody RL2. The expressed HA-AKT in the cell extracts (Ext) and the immunoprecipitates (IP) were then determined by Western blots developed with anti-HA and RL2. (**F**) The expressed AKTs of the cell extracts were also immunoprecipitated using anti-HA antibody, followed by Western blots developed with RL2 and anti-HA. The arrow indicates the position of AKT band, and the arrowhead indicates the heavy chain of IgG.

Next, we investigated the possible mechanism by which O-GlcNAcylation promotes apoptosis. Because AKT signaling, which is regulated by O-GlcNAcylation^15^,^16^, is known to promote growth factor–mediated cell survival and to inhibit apoptosis via phosphorylating the pro-apoptotic protein Bad at Ser136^17^, we investigated AKT phosphorylation at Thr308 and Ser473, which determines AKT’s kinase activity^18^, and Bad phosphorylation after manipulation of O-GlcNAcylation level in HEK-293FT cells. As expected, over-expression of OGT and OGA led to marked increase and decrease, respectively, of cellular protein O-GlcNAcylation levels, as determined by Western blots developed with monoclonal antibody RL2 (Fig. 2A). We found that the increase of O-GlcNAcylation, as the result of OGT over-expression, resulted in a decrease in phosphorylation of both AKT and Bad at the activity-relevant sites. In contrast, reduction of O-GlcNAcylation, as the result of OGA over-expression, resulted in an increase in AKT and Bad phosphorylation. These results suggest that O-GlcNAcylation might promote apoptosis through down-regulating AKT activity, which in turn leads to less phosphorylation of Bad at Ser136 and thus increase in pro-apoptotic activity.

To verify the key role of AKT in cell apoptosis, we knocked down AKT with its siRNA or inhibited its activity with LY294002 in HEK-293FT cells and investigated three apoptotic markers. We found that either AKT knock-down (evidenced by the AKT blot) or inhibition (evidenced by reduction of its phosphorylation at Ser473) resulted in a reduction of Bad phosphorylation at Ser136 and an increase in the cleavages of caspase 3 and PARP (Fig. 2B,C). These results further support that an O-GlcNAcylation-induced decrease of AKT phosphorylation/activation could lead to cell apoptosis.

It has been shown that AKT itself is modified by O-GlcNAc^15^,^19^,^20^,^21^, but the exact O-GlcNAcylation sites are unknown. The negative regulation of AKT phosphorylation at Thr308 and Ser473 by O-GlcNAcylation observed above suggests that these two residues might be modified by O-GlcNAc. To elucidate if this is the case, we investigated the O-GlcNAcylation of WT-AKT and mutant AKT in HEK-293FT cells. We first over-expressed WT-AKT and mutant AKT in which Thr308 and/or Ser473 were mutated to alanine, so that these amino acid residues could no longer accept O-GlcNAc or phosphate modification. Stimulation of AKT signaling in the cultured cells with insulin after serum withdrawal was used to verify the AKT mutant expression. As expected, phosphorylation of AKT at both Thr308 and Ser473 was seen in WT-AKT-transfected, but not at Thr308 in AKT_T308A_-transfected or at Ser473 in AKT_S473A_-transfected cells (Fig. 2D). Phosphorylation of AKT at neither site was found in AKT_2A_. These results confirmed the expression of the specific AKT mutants in HEK-293FT cells.

To investigate whether Thr308 and Ser473 of AKT are modified by O-GlcNAc, we transfected HEK-293FT cells with HA-tagged WT-AKT and its mutants in combination with OGT. The O-GlcNAcylated proteins of the cell lysates were then immunoprecipitated by using monoclonal antibody RL2 and analyzed by Western blots. As shown in Fig. 2E, OGT transfection led to a marked increase in protein O-GlcNAcylation, and the O-GlcNAcylated proteins were successfully immunoprecipitated with RL2. When anti-HA was used to detect HA-AKT in the immunoprecipitates, we found much more WT-AKT and AKT_S473A_ with OGT transfection as compared to those without OGT transfection (Fig. 2E), indicating that the over-expressed OGT led to AKT O-GlcNAcylation. It was also clear that mutation of Ser473 and, more markedly, of Thr308 into alanine reduced the amount of O-GlcNAcylated AKT, as compared with WT-AKT. Double mutations at both residues completely eliminated AKT O-GlcNAcylation. Similar results were obtained when the cell lysates were immunoprecipitated with anti-HA and then the immunoprecipitates were detected by using RL2 (Fig. 2F). These results indicate that AKT can be modified by O-GlcNAc at both Thr308 and Ser473.

### AKT over-expression attenuates O-GlcNAcylation-induced apoptosis

**Figure 3:**
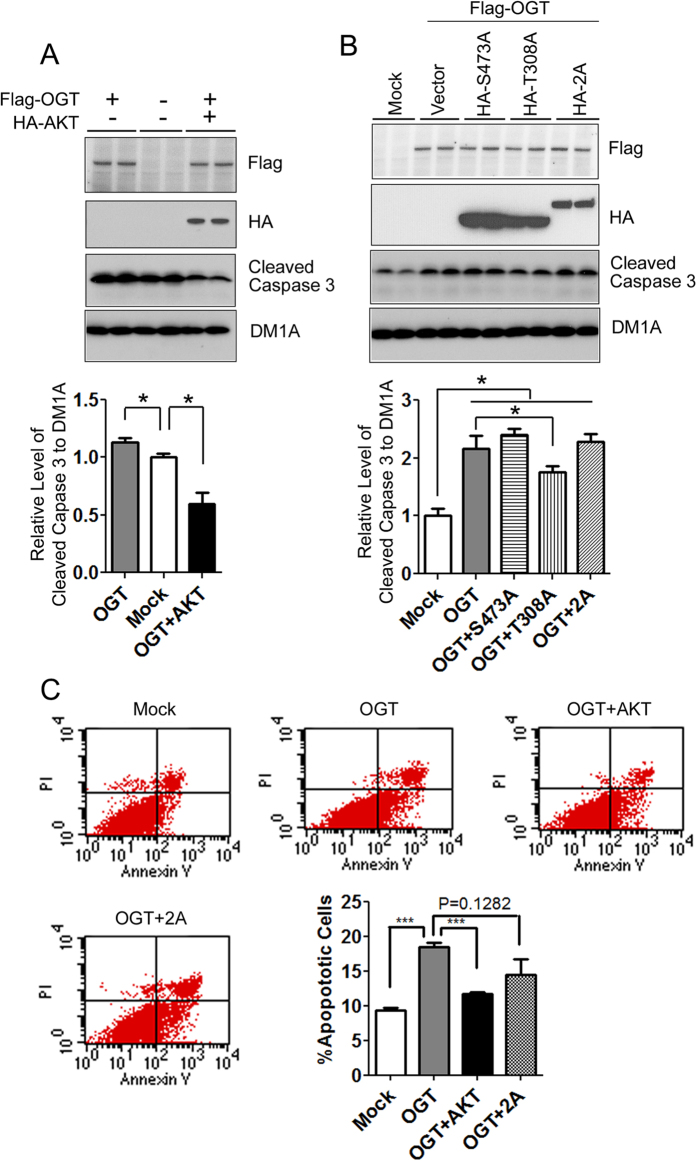
AKT over-expression attenuates OGT-induced apoptosis. (**A**,**B**) HEK-293FT cells were transfected with Flag-OGT with or without co-transfection with HA-AKT or its mutants for 48 h, and the cleaved caspase 3 in the cell lysates was determined by Western blots. Densitometrical quantifications of the blots after normalization with tubulin (DM1A blot) are shown in the graphs. (**C**) The transfected cells were also stained with Annexin V and PI and analyzed in a flow cytometer. The percentages of apoptotic cells (in the lower right part) were quantified and are shown in the graph. Data are presented as mean ± SD (n = 6). *p < 0.05, ***p < 0.001 vs. the mock group.

Besides AKT, other components of the PI3K-AKT signaling pathway are also modified by O-GlcNAcylation^20^. To confirm whether AKT is a critical mediator through which O-GlcNAcylation promotes apoptosis, we co-transferred HEK-293FT cells with AKT and OGT and found that AKT expression prevented OGT-induced apoptosis, as indicated by the level of cleaved caspase 3 (Fig. 3A). When AKT mutants were used to replace the wild type AKT, the preventive role was seen only with AKT_T308A_, but not with AKT_S473A_ although its expression was a little bit higher than that of AKT_T308A_, as determined by HA blots (Fig. 3B). These results suggest that Ser473 of AKT is required for the preventive activity against OGT-induced apoptosis. Although a lower expression of AKT_2A_ than the single mutants was obtained under our conditions (Fig. 3B), the lack of preventative activity of AKT_2A_ against OGT-induced apoptosis is consistent to our conclusion of the requirement of Ser473 of AKT for its preventive activity.

We also analyzed cell apoptosis induced by OGT over-expression with and without AKT co-transfection by using flow cytometry. As expected, OGT transfection resulted in marked increase in cell apoptosis, and co-transfection with AKT prevented OGT-induced apoptosis (Fig. 3C). When the cells were co-transfected with OGT and inactive AKT_2A_, no significant prevention was observed. These results confirmed that O-GlcNAcylation promotes cell apoptosis through modulation of AKT signaling.

### Protein O-GlcNAcylation level is altered dynamically in the mouse brain after MCAO

**Figure 4:**
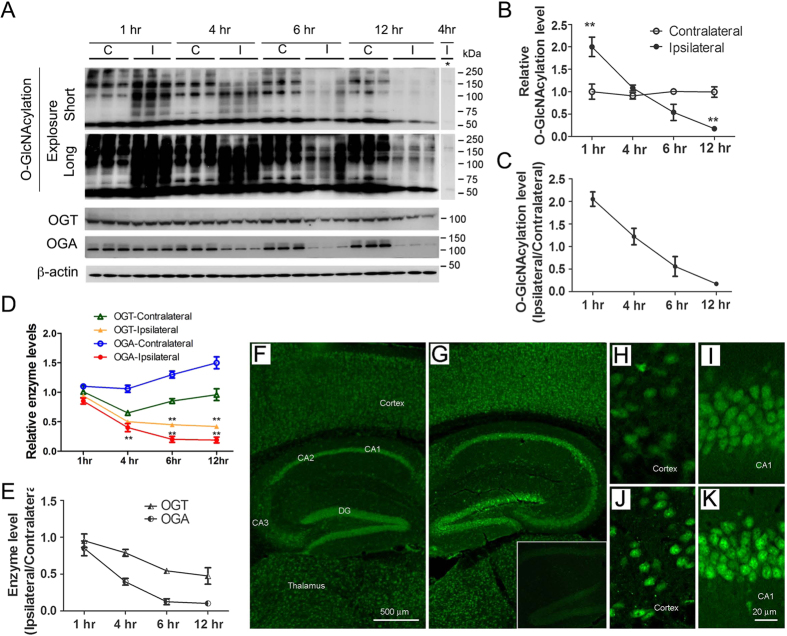
Time-dependent alterations of protein O-GlcNAcylation and its catalyzing enzymes in the cerebral cortex of mouse brain after MCAO. (**A**) Mice were sacrificed at the indicated time points after MCAO, and the levels of protein O-GlcNAcylation, OGT, OGA, and β-actin (as a loading control) in the homogenates of the ipsilateral (**I**) and contralateral (**C**) cortices were determined by Western blots developed with antibodies indicated at the left of the blots. Asterisk (*) on the top of the right most lane indicates that the blot was incubated with the primary antibody (RL2) solution after addition of 200 mM GlcNAc and pre-incubation at room temperature for 30 min. (**B**–**E**) Densitometric quantification of the blots as shown in panel A. The relative levels (mean ± SD, n = 5), where the levels of the contralateral cortex at 1 h were set to be 1.0 (**B**,**D**), and the ratio of ipsilateral over contralateral sides (**C**,**E**) are shown. **p < 0.01 vs. contralateral side. (**F**–**K**) Immunofluorescence staining of O-GlcNAcylation in the contralateral (**F**,**H**,**I**) and ipsilateral (**G**,**J**,**K**) brains of mice 1 h after MCAO. Frozen sections were immunostained with a mixture of two monoclonal antibodies (RL2 and CTD110.6), which both recognize O-GlcNAcylated proteins. As a negative control staining (**G**, insert), before the ipsilateral section was incubated with the antibodies, 200 mM GlcNAc was added into the primary antibody solution followed by pre-incubation of the antibody solution at room temperature for 30 min.

**Figure 5:**
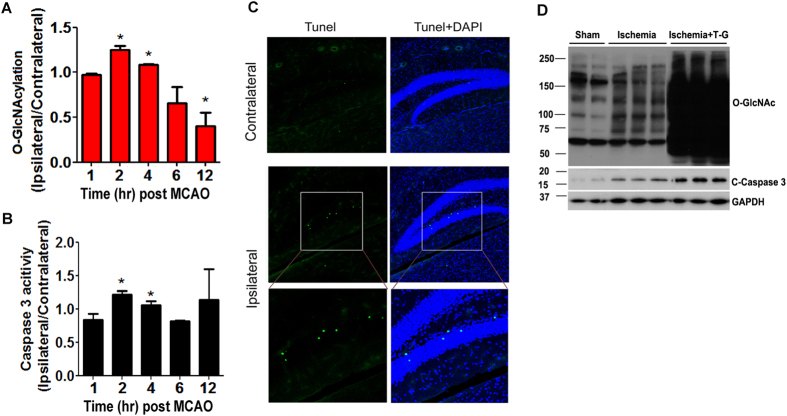
Dynamic alterations of ischemia-induced protein O-GlcNAcylation and apoptosis in the mouse hippocampus. (**A**) Mice were sacrificed at the indicated time points after MCAO, and the level of protein O-GlcNAcylation in the hippocampus were determined by quantitative Western blots. (**B**) Caspase 3 activity in the hippocampal extract was also measured. Data are presented as mean ± SD (n = 5). *p < 0.05 vs. controls. (**C**) Frozen brain sections from the mice sacrificed 2 h post MCAO were double-labeled with TUNEL staining (green) for apoptotic cells and DAPI (blue) for the nuclei. (**D**) Mice were administered with 160 μg thiamet-G (T-G) or, as a control, saline through icv injection, followed by MCAO 24 h later. The mice were then sacrificed 2 h after MCAO, and the levels of O-GlcNAcylation and cleaved/activated caspase 3 (C-caspase 3) in the ipsilateral cerebrocortical homogenates were determined by Western blots. GAPDH blot was included as a loading control.

To learn the role of O-GlcNAcylation in regulating neuronal apoptosis, we investigated O-GlcNAcylation and apoptosis in a mouse model of MCAO. In the MCAO model, the striatum is usually the core of ischemia, leading to irreversible damage and necrosis, whereas reversible damage usually occurs in the adjacent cerebral cortex, and the hippocampus is less affected. We therefore investigated protein O-GlcNAcylation in the cerebral cortex and the hippocampus during ischemia induced by MCAO. We observed dynamic changes of global protein O-GlcNAcylation both in the cerebral cortex and the hippocampus after MCAO. In the ipsilateral cerebral cortex, the global protein O-GlcNAcylation level increased remarkably, as determined by Western blots developed with monoclonal antibody RL2 against O-GlcNAcylated proteins, one hour after MCAO (Fig. 4A–C). However, the O-GlcNAcylation level quickly decreased afterwards and reached a nearly undetectable level 12 h after MCAO. The O-GlcNAcylation level on the contralateral cerebral cortex, as the control, did not change significantly throughout this period of time (Fig. 4A–C). Similar dynamic changes of global O-GlcNAcylation, which occurred to a less extent and delayed dynamics, were seen in the ipsilateral hippocampus (see Fig. 5A below). The RL2 immuno signal was specific to O-GlcNAc because, as a control blot, addition of GlcNAc (200 mM) into the primary antibody solution blocked the immunoreactivity almost completely.

Along with the marked dynamic changes of O-GlcNAcylation, massive downshifts of the O-GlcNAcylated protein bands of the ischemic (ipsilateral) brain tissue were seen (Fig. 4A). These downshifts of molecular weights are probably resulted from ischemia-induced partial proteolysis of cellular proteins. It is well known that cerebral ischemia causes calcium overload and release of lysosomal enzymes, which in turn lead to cellular protein proteolysis. It is also clear that not all cellular proteins are equally vulnerable to proteolysis under the ischemic condition, because β-actin was not proteolyzed, whereas OGA was rapidly degraded, under the same ischemic conditions (Fig. 4A).

We also determined the levels of OGT and OGA, which together control the O-GlcNAcylation level. We found a time-dependent decrease in both OGT and OGA levels in the ipsilateral cerebral cortex after MCAO (Fig. 4A,D,E). The decrease in OGA level was very dramatic after MCAO. There were no significant changes in the OGT or OGA levels in the contralateral control side, although the OGA level appeared to have increased 6–12 h after the surgery. The time-dependent decreases of OGT and OGA did not coincide exactly with the dynamic changes of O-GlcNAcylation levels (up at 1 hr post ischemia and then down afterwards) in the ipsilateral side of the brain, which could otherwise explain the transient increase of global O-GlcNAcylation within four hours after cerebral ischemia.

To learn the topographic distribution of elevated O-GlcNAcylation in the ischemic brain, we immunostained the frozen brain sections from mice after MCAO with a mixture of two monoclonal antibodies (RL2 and CTD110.6), which both recognize various O-GlcNAcylated proteins. We observed a marked increase in the immunostaining at the ischemic side (Fig. 4G) as compared to the contralateral control side (Fig. 4F) of the brain 1–2 h after MCAO. The fluorescence signal was specific to O-GlcNAc because only background fluorescence was seen when GlcNAc (200 mM) was added into the primary antibody solution (Fig. 4G, insert). The increased O-GlcNAc immunostaining was obvious in the cerebral cortex and hippocampus (Fig. 4G). In the hippocampus, marked increase in O-GlcNAc staining was seen in the pyramidal neurons of all sectors of the cornu Ammonis (CA) and the granular neurons of the dentate gyrus (DG) of the ipsilateral side (Fig. 4G) as compared to the contralateral side (Fig. 4F). High magnification demonstrates clearly both nuclear and cytoplasmic distribution of the neuronal O-GlcNAcylated proteins (Fig. 4H–K).

### Elevation of protein O-GlcNAcylation coincides with activation of apoptosis in the mouse brain after MCAO

Previous studies had suggested that O-GlcNAcylation might regulate apoptosis, and that apoptosis plays an important role in ischemia-induced neuronal death^1^. Therefore, we investigated whether the dynamic changes of O-GlcNAcylation coincide with activation of apoptosis in the mouse brain after MCAO. We observed a good match between the dynamic elevation of O-GlcNAcylation and apoptosis in the mouse brain. In the hippocampus, both the elevation of O-GlcNAcylation and apoptosis, as evidenced by caspase 3 activation, peaked 2 h after MCAO (Fig. 5A,B). Very large individual variations of caspase 3 activity were seen in the mouse hippocampi 12 h after MCAO, but the mean value was not significantly different from that of the sham surgical group. Cell apoptosis was further confirmed in the brains of mice 2 h after MCAO by using TUNEL staining. We observed apoptotic cells in the ipsilateral brain side, but not in the contralateral brain side, after MCAO. The apoptotic cells were most evident in the dentate gyrus of the hippocampus (Fig. 5C). More cell apoptosis, as evidenced by more caspase 3 cleavage/activation (Fig. 5D), was observed in the ipsilateral brain of MCAO mice if protein O-GlcNAcylation level was elevated by an icv injection of thiamet-G (160 μg/mouse), a potent OGA inhibitor, 24 h before MCAO. These results together support a role of the elevated O-GlcNAcylation in cell apoptosis during ischemia.

### O-GlcNAcylation level correlates negatively with AKT phosphorylation in ischemic brain tissue

**Figure 6:**
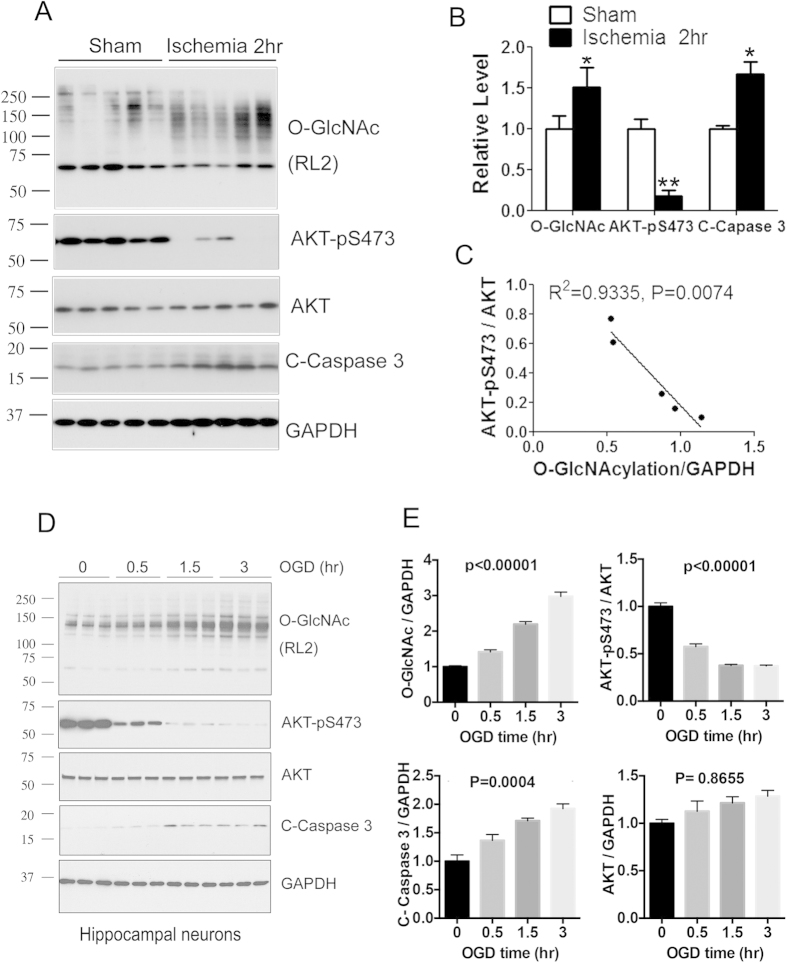
O-GlcNAcylation negatively correlates with AKT phosphorylation in the ischemic brain tissue of MCAO mice and in cultured neurons. (**A**) Western blots of cerebrocortical homogenates from sham or MCAO mice 2 h after surgery. (**B**) Densitometrical quantifications of AKT-pS473 after normalization by the AKT level and of O-GlcNAcylation and cleaved caspase 3 levels after normalization by the GAPDH level. Data are presented as mean ± SD (n = 5). *p < 0.05 vs. sham. **p < 0.01 vs. sham. (**C**) The O-GlcNAcylation levels were plotted against the level of AKT-pS473/AKT in the hippocampal homogenates from MCAO mice (n = 5). (**D**) Hippocampal neurons were cultured in medium deprived of oxygen and glucose (OGD) for up to 3 hrs, followed by Western blots of the cell lysates developed with the antibodies indicated on the right side of the blots. (**E**) Densitometrical quantifications of the blots from panel D. Data are presented as mean ± SD (n = 3). One-way ANOVA was used to calculate the statistical significance.

To investigate whether O-GlcNAcylation promotes neuronal apoptosis through inhibition of AKT phosphorylation/activation in ischemic brain, we determined the levels of O-GlcNAcylation, AKT phosphorylation and caspase 3 cleavage in ischemic and control (sham) brain tissue of mice and analyzed the relationship between O-GlcNAcylation and AKT phosphorylation. As shown above, marked increase in O-GlcNAcylation and caspase 3 cleavage, as well as marked decrease in AKT phosphorylation at Ser473 were observed in the ischemic tissue, as compared to the sham control (Fig. 6A,B). The basal level of Thr308 phosphorylation of AKT in the mouse brain was too low for reliable determination under these conditions. Correlation analysis indicated a strong negative relationship between O-GlcNAcylation and AKT phosphorylation at Ser473 in MCAO mice (Fig. 6C). These results support that the ischemia-induced elevation of O-GlcNAcylation may down-regulate AKT phosphorylation/activation and thus promote apoptosis in the brain *in vivo*.

To mimic the cerebral ischemic injury, we deprived oxygen and glucose from the medium of cultured hippocampal neurons for up to 3 hrs and studied the levels of O-GlcNAcylation, AKT, phospho-AKT, and caspase 3 cleavage. We found an elevated O-GlcNAcylation level (as detected with RL2), decreased AKT phosphorylation and increased caspase 3 cleavage 3 hrs after oxygen and glucose deprivation (Fig. 6D,E). These results further support our conclusion that O-GlcNAcylation promotes neuronal apoptosis through down-regulation of AKT activity under ischemic conditions.

## Discussion

Neuronal cell death during cerebral ischemia is crucial to the pathological process and prognosis of patients suffering from cerebrovascular disorders, such as ischemic stroke. Both necrosis and apoptosis may occur in the process of cerebral ischemia. Apoptosis appears in the early stages of ischemia. With the extension of ischemic time and the expansion of ischemic area, a large number of cells undergo necrosis in the central area of ischemia, and apoptotic cells are often found around the penumbra^22^,^23^. Necrosis is usually caused by factors external to the cell or tissue, such as infection, toxins and trauma, which results in unregulated digestion of cell components and leads to severe localized inflammatory response. In contrast to necrosis, apoptosis is usually an orderly process of energy-dependent programmed cell death to dispose of redundant cells, which involves no inflammation and minimizes damage and disruption to the neighboring cells. In the present study, we found that O-GlcNAcylation induced apoptosis in cultured cells. Investigation into its mechanism indicated that O-GlcNAcylation promotes cell apoptosis likely through modification of AKT at Ser473 and Thr308 and of Bad at Ser136 with O-GlcNAc and thus attenuation of their phosphorylation at these sites. In addition, AKT over-expression attenuated O-GlcNAcylation-induced apoptosis. Using the mouse MCAO model, we found a dynamic elevation of brain protein O-GlcNAcylation which coincided with the activation of neuronal apoptosis at the early ischemic stage (within four hours). In the ischemic brain tissue the O-GlcNAcylation level correlated negatively with AKT phosphorylation. These studies reveal, for the first time, an important regulation of ischemia-induced neuronal apoptosis by O-GlcNAcylation involving AKT signaling.

Many studies have shown that apoptosis is a double-edged sword in cerebral ischemia. During the early stage of acute ischemia, apoptosis is likely a self-protective response to the environment of oxygen and glucose deprivation, which helps the maintenance and survival of the most vital cells. With extensive period of ischemia, however, calcium overload, oxygen free radicals and release of lysosomal enzymes lead to necrosis of cells^24^. Early apoptotic response might help limit inflammatory and immune responses that are otherwise toxic and deteriorative to the surrounding cells, causing lesion expansion^25^. Our observations from the present study suggest the dynamic elevation of brain O-GlcNAcylation, which promotes apoptosis, may be beneficial to restriction of ischemia-induced brain damage. These findings are consistent to a recent study showing that intraperitoneal administration of glucosamine, which increases protein O-GlcNAcylation, reduces infarct size, motor impairment and neurological deficits in rats through reduction of neuroinflammation in a MCAO model^26^. The protective role of O-GlcNAcylation in myocardial ischemic injury has been reported previously. In a myocardial ischemia-reperfusion model, elevation of protein O-GlcNAcylation with glucosamine almost completely eliminates myocardial injury induced by calcium overload and ischemic stress^8^. PUGNAc, an inhibitor of OGA, increases O-GlcNAcylation levels and reduces the area of myocardial infarction^9^. The present study indicates that the protective role of O-GlcNAcylation may not be restricted in myocardial ischemia.

We have observed that a robust and widespread increase in O-GlcNAcylation level was accompanied with only sporadic positive TUNEL staining in the ischemic brain. This phenomenon probably suggests a high tolerance of mammalian brains to the elevation of O-GlcNAcylation. Indeed, a marked increase in brain O-GlcNAcylation does not result in any detectable brain or behavioral abnormalities in mice^27^,^28^. Our findings that up-regulation of O-GlcNAcylation promoted cell apoptosis are consistent with several previous reports. Liu *et al.* observed neuronal apoptosis in brain areas with high O-GlcNAcylation^11^. Elevation of O-GlcNAcylation may cause pancreatic β-cell apoptosis^11^,^12^. In a human prostate cancer cell line (ALVA41), O-GlcNAcylation promotes apoptosis by the inhibition of proteasome^29^. In myocardial cells, O-GlcNAcylation is shown to regulate Bad and Bcl-2 and causes apoptosis^13^. Induction of mitochondrial OGT expression is also shown to promote cell apoptosis^14^. In contrast, a possible anti-apoptotic action of O-GlcNAcylation has also been reported in the myocardial ischemia-reperfusion model because glucosamine-induced elevation of O-GlcNAcylation induces expression of the anti-apoptotic gene Bcl-2^8^. However, the study did not rule out the possibility that the Bcl-2 expression could have resulted from a negative feedback of the O-GlcNAcylation-induced apoptosis.

**Figure 7:**
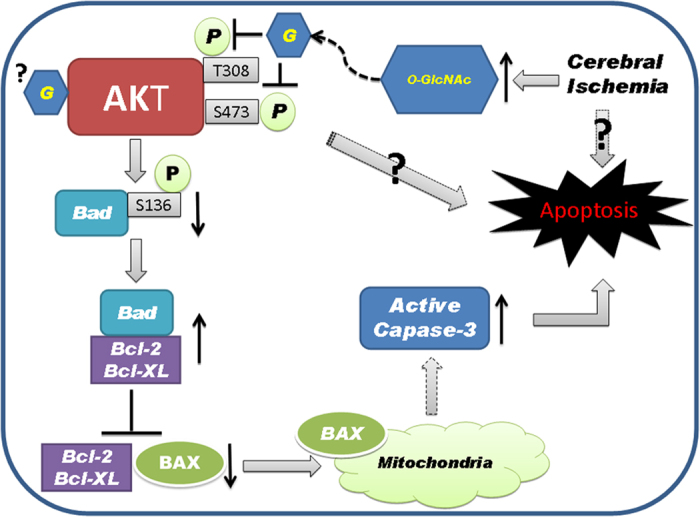
Proposed mechanism by which O-GlcNAcylation promotes cell apoptosis. O-GlcNAc modifies AKT at both Thr308 and Ser473 and thus attenuates its phosphorylation/activation, which is a well-known key regulator of cell death and survival. The down-regulation of AKT would result in a decrease in phosphorylation of Bad, a pro-apoptotic protein, and thus increase its activity to bind to and to inhibit the pro-survival Bcl-2 family proteins, because Bad phosphorylation leads to inactivation of its activity. As the consequence, the Bax release would increase and attack mitochondrial membrane, leading to cytochrome C release and caspase-3 activation, and finally triggers apoptosis.

In the present study, we further investigated the molecular mechanism by which O-GlcNAcylation induces apoptosis. Because O-GlcNAcylation negatively regulates the activation of AKT signaling^15^, which is a well-known key regulator of cell death and survival^30^,^31^, we investigated the effects of O-GlcNAcylation on the phosphorylation/activation of AKT. We found that O-GlcNAc appears to modify AKT at both Thr308 and Ser473, which occupies the hydroxyl group of these two residues and thus makes them unavailable for phosphorylation. Consequently, O-GlcNAcylation attenuates AKT phosphorylation/activation. The down-regulation of AKT could result in a decrease in phosphorylation of Bad, a pro-apoptotic protein, and thus increase its activity to bind to and to inhibit the pro-survival Bcl-2 family proteins (Fig. 7), because Bad phosphorylation leads to inactivation of its activity^17^. As the consequence, the Bax release would increase and attack mitochondrial membrane, leading to cytochrome C release and caspase-3 activation, and finally triggers apoptosis. Increased O-GlcNAcylation could also result in decreased phosphorylation of Bad directly, because it has been shown in myocardial cells that increased global O-GlcNAcylation increases Bad O-GlcNAcylation, enhances its interaction with Bcl-2, and leads to apoptosis^13^.

O-GlcNAcylation is regarded to be a sensor of intracellular glucose metabolism because OGT activity is mainly regulated by intracellular concentration of UDP-GlcNAc that is the product of glucose metabolism through the hexosamine biosynthetic pathway^32^. Under ischemic condition, it is reasonable to expect a reduction of global O-GlcNAcylation due to decreased glucose metabolism. However, we observed marked elevation of global protein O-GlcNAcylation instead in the mouse brain within four hours after MCAO. Because there was no concurrent elevation of OGT or decrease of OGA, this dynamic increase in O-GlcNAcylation might be caused by an acute stress response of the brain to severe ischemia. Marked reduction of O-GlcNAcylation in the ischemic brain tissue after ischemia for four hours or longer supports our speculation of temporary response. Such a response is consistent with previous reports showing increased protein O-GlcNAcylation after deprivation of glucose in neuronal N2a cells^33^ and in liver HepG2 cells^34^,^35^. Future studies on the dynamic changes of intracellular UDP-GlcNAc level and the activity of GFAT, the rate-limiting enzyme of the hexosamine biosynthetic pathway, will help elucidate the molecular mechanism underlying the dynamic elevation of protein O-GlcNAcylation during the early stage of cerebral ischemia.

Following the marked temporary increase, we observed a deep reduction of protein O-GlcNAcylation starting after four hours post MCAO, with the level nearly undetectable 12 hours after cerebral ischemia. The reduction of cerebral protein O-GlcNAcylation is likely due to the exhaustion of intracellular UDP-GlcNAc from depletion of glucose metabolism. Cellular death caused by sustained apoptosis and necrosis at the longer time points might also contribute to the reduction of protein O-GlcNAcylation, as the levels of OGT and OGA, especially of the latter, as well as of some other proteins (data not shown), were found to be decreased in a time-dependent manner, suggesting overall increase of protein degradation at the second phase of cerebral ischemia.

In conclusion, we show here, for the first time, the dynamic alteration of cerebral protein O-GlcNAcylation during cerebral ischemia in the mouse model of MCAO and demonstrate an important role of O-GlcNAcylation in the regulation of ischemia-induced neuronal apoptosis. These studies provide a novel insight into the molecular mechanisms involved in the severity of ischemia-induced brain injury. Elucidation of the role of O-GlcNAcylation in cerebral ischemia might lead to the identification of a novel therapeutic target for the clinical management of stroke and other ischemic cerebrovascular diseases.

## Materials and Methods

### Reagents and Antibodies

LY294002, DAPI, antibodies against β-actin, α-tubulin (DM1A), OGT and HA were products of Sigma-Aldrich Corp. (St. Louis, MO, USA). Antibody against Glyceraldehyde-3-phosphate dehydrogenase (GAPDH) was the product of Santa Cruz Biotechnology Inc (Santa Cruz, CA, USA). Antibodies against AKT, AKT-pS473, AKT-pT308, Bad, p-Bad (Ser136), Cleaved Caspase3, Caspase3, cleaved PARP were purchased from Cell Signaling Technology, Inc. (Danvers, MA, USA). ECL kit, protein G agarose beads, and antibody against O-GlcNAc-modified proteins (RL2) were purchased from Thermo Fisher Scientific (Rockland, IL, USA). Antibody against OGA was a gift from S. W. Whiteheart (University of Kentucky College of Medicine, Lexington, KY). Peroxidase-conjugated anti-mouse and anti-rabbit IgG were obtained from Jackson ImmunoResearch Laboratories (West Grove, PA). Alexa 488- conjugated goat anti-mouse IgG, and TO-PRO-3 iodide (642/661) were from life Technologies (Grand Island, NY, USA). CellTiter 96® Non-Radioactive Cell Proliferation Assay and DeadEnd™ Fluorometric TUNEL kits were from Promega (Madison, WI, USA). Caspase 3 Assay Kit (Colorimetric) was from Abcam (Cambridge, MA, USA).

### Plasmid Construction and DNA Mutagenesis

Mammalian expression vector pCMV-Entry-hOGT1 was bought from Origene Company (Rockville, MD). The vector pHRST-OGT-IRES-GFP was subcloned from pCMV-Entry-hOGT1. The expression constructs for human OGA and AKT were generated by reverse-transcription PCR from RNA isolated from normal human neuronal progenitor cells and were confirmed by DNA sequence analysis. AKT tagged with hemagglutinin (HA) was cloned into the pCI-Neo vector via the XhoI and Sal I sites. Mutation of AKT at Thr-308 or Ser-473 to Ala was achieved by using the Quick Change II site-directed mutagenesis kit (Stratagene, La Jolla, CA) with the following forward and reversed primers: 5′-ggtgccaccatgaagGccttttgcggcacacctg-3′ and 5′-caggtgtgccgcaaaaggCcttcatggtggcacc-3′ for Thr-308 to Ala, and 5′-ccacttcccccagttcGcctactcggccagcggc-3′ and 5′-gccgctggccgagtaggCgaactgggggaagtgg-3′ for Ser473 to Ala. The mutation at Thr-308 and Ser-473 to Ala (AKT2A) were produced by using PCR from vector pCI-Neo-HA AKT T308A with the same forward primers used for Ser-473 to Ala. The mutations were confirmed by DNA sequencing.

### Cell culture, transfection, and Cell viability Assays

Human embryonic kidney cell line (HEK-293FT) and Human cervix epithelia cell line (Hela) were cultured in Dulbecco’s modified Eagle’s medium supplemented with 10% fetal bovine serum, 100 U/ml penicillin, and 100 U/ml streptomycin and incubated in a humidified atmosphere containing 5% CO_2_ at 37 °C.

Transfection of HEK-293FT cells were performed using FuGENE 6 (Roche) in 12-well plates. HEK293FT cells were transfected with 0.5 μg of plasmid DNA and 1.5 μl of FuGENE 6 for 48 hr. Lipofectamine^TM^2000 reagent (Invitrogen) was used for the transfection of Hela cells according to the manufacturer’s instruction. For knock-down of OGT expression, we used SureSilencing shRNA plasmids for human OGT (SA Bioscience, Frederick, MD). They were designed to target CCAAGGACGATACTGAAAGTT (shOGT1) and AGATCTTCGAACAGCCAGAAT (shOGT2) of the human OGT under the control of the U1 promoter and the GFP gene. shRNA with the sequence of CCATCGCCAAGCTGATTAAAT was used to be a negative control. For inhibition of AKT1 expression, HEK-293FT cells cultured in 12-well plates were transfected with various amounts of short interfering RNA (siRNA, Santa Cruz Technology) using Lipofectamine 2000.

After HEK293FT cells were transfected with OGT or shOGT for 48 hours in 96-well plates, cell viability was measured by a colorimetric assay with CellTiter 96® Non-Radioactive Cell Proliferation Assay according to the manufacturer’s instruction.

Mouse primary hippocampal neuronal cells were prepared from hippocampi of embryonic C57B6 mice (E16). Cells were plated (50,000 cells/m^2^) in poly-D-lysine–coated flasks (BD Falcon, Franklin Lakes, NJ) and cultured in Neurobasal medium containing 2% B-27 supplement, 50 μg/ml gentamicin, and 2 mM L-glutamine (Invitrogen). Cultures were kept at 37 °C in a moist atmosphere (5% CO_2_). Half of the medium was replaced by fresh medium every 3 days, and 7-day-old cultures were used for oxygen/glucose deprivation (OGD) study. The culture medium was replaced by glucose-free Earle’s balanced salt solution purged by nitrogen gas for 10 min (pO^2^ ≈ 5–6%). Then the cells were placed for 0.5, 1.5, or 3 h in a chamber filled with 5% CO_2_ and 95% N_2_ before being harvested for Western blot analysis.

### Mouse transient middle cerebral artery occlusion (MCAO)

Male ICR mice weighing 25–30 g were purchased from Shanghai SLAC Laboratory Animal CO. Ltd (Shanghai, China). The use of mice was carried out in accordance with the recommendations in the Guide for the Care and Use of Laboratory Animals of the National Institutes of Health (USA). The animal use protocol was approved by the Committee on the Ethics of Animal Experiments of Nantong University. All surgeries were performed under anesthesia with pentobarbital (40 mg/kg), and efforts were made to minimize suffering of the animals. The mice were fasted overnight but were allowed free access to water before surgical procedure. A heating pad and a heating lamp were used to maintain the rectal temperature between 36.5 °C and 37.5 °C. Through a ventral midline incision, the right common carotid artery, internal carotid artery and external carotid artery were surgically exposed. A 6–0 nylon suture with silicon coating (Doccol Corporation, Redlands, USA) was inserted into the internal carotid artery through the external carotid artery stump and gently advanced to occlude the middle cerebral artery. Laser-Doppler flowmetry (LDF, ML191 Laser Doppler Blood FlowMeter, Australia) was used to monitor the blockade of cerebral blood flow of the middle cerebral artery territory. Then, the neck incision was closed, and the mice were allowed to recover. The body temperature of each mouse was carefully monitored during the post-operation period until the complete recovery from anesthesia. After surgery, the animals were housed individually until euthanized. All animals had free access to food and water. At different time points after MCAO procedure, the mice were sacrificed, and the brains were immediately removed and either processed for fluorescent immunohistochemistry and TUNEL staining or homogenized at 4 °C in a buffer containing 50 mM Tris–HCl, pH 7.4, 150 mM NaCl, 50 mM NaF, 1 mM Na_3_VO_4_, 1% Triton X-100, 1% NP40, 0.25% sodium deoxycholate and the Roche complete mini protease inhibitor cocktail. The homogenates were used for Western blots.

Some mice received intracerebroventricular (icv) injection of 160 μg/mouse thiamet-G (Cayman Chemical, Ann arbor, MI) dissolved in 3 μl saline or, as a control, saline alone 24 h before MCAO. For icv injections, the anesthetized mice were restrained onto a stereotaxic apparatus. Each mouse received a single icv injection into the right lateral ventricle slowly over a period of 5 min, and the needle was retained for another 10 min before removal. The bregma coordinates used for icv injection were: −1.0 mm lateral, −0.3 mm posterior, and −2.5 mm below.

### Western blots

For Western blots, cultured cells were lysed in Laemmli SDS sample buffer directly, and brain homogenates were diluted in 2 × Laemmli SDS sample buffer at 1:1 ratio, followed by heating at 95 °C for 5 minutes. Samples were first resolved in 10% SDS-PAGE, and the proteins in the gels were transferred onto Immobilon membrance (Milipore), followed by incubation with primary antibody, washing and then with HRP-conjugated secondary antibodies. The protein-antibody complex were visualized by the Pierce ECL Western Blotting Substrate (Thermo scientific) and exposed to Kodak medical X-ray film (Kodak, USA). Specific immunostaining was quantified by using the Multi Gauge software V3.0 from Fuji Film. Each blot was exposed for 3–4 different periods of time, and only those with adequate exposure were used for quantification.

### Immunoprecipitation

Cultured cells were washed twice with PBS and then lysed in the lysate buffer (50 mM Tris-HCl, pH 7.4, 150 mM NaCl, 50 mM NaF, 1 mM Na_3_VO_4_, 1% Triton X-100, 1% NP40, 0.25% sodium deoxycholate and the Roche complete mini protease inhibitor cocktail). Insoluble materials from mouse hippocampal homogenates and cultured cell lysates were removed by a brief centrifugation at 4 °C, and the supernatants were incubated with the immunoprecipitating antibody pre-coupled onto protein G beads at 4 °C for 4 hr. The beads were washed with the lysate buffer twice and then with TBS twice. The bound proteins were eluted by boiling the beads in Laemmli sample buffer. The samples were analyzed by Western blots.

### Fluorescent immunohistochemistry

Frozen sections (40-μm thick) were first blocked with 5% normal goat serum in TBS for 30 min, followed by incubation with a primary antibody overnight at 4 °C in TBS containing 5% goat serum and 0.1% Triton. After washing with TBS, the sections were incubated with Alexa 488-conjugated goat anti-mouse IgG (1:1000) plus DAPI in TBS containing 5% goat serum and 0.1% Triton at room temperature for 1 h. The immunostaining was analyzed by using a laser scanning confocal microscope (PCM 200, Nikon).

### Caspase-3 activity assay

Frozen mouse brain tissue was homogenized in 9 volumes of chilled cell lysis buffer (see above) in a Dounce homogenizer for around 40 passes on ice, followed by centrifugation at 10,000 × *g* in a microcentrifuge for 1 min. The supernatant was collected for measuring protein concentration with modified lowery method and caspase-3 assay. For the latter, 100 μg protein of each sample was diluted with 50 μl cell lysis buffer for each assay, followed by adding 50 μl of 2 × reaction buffer (containing 10 mM DTT). Into each sample 5 μl of 4 mM DEVD-p-NA substrate (200 μM final conc.) was added and incubated at 37 °C for 2 hr. Optical density was measured at 405 nm in a microtiter plate reader.

### TUNEL staining

Frozen brain sections were washed twice in PBS and then permeabilized with 20 μg/ml proteinase K for 10 min. After washing again in PBS for 5 min, brain sections were fixed in 4% formaldehyde in PBS for 5 min. After washing and equilibration at room temperature for 5–10 min, TdT reaction mix was added, followed by incubation at 37 °C in a dark humidified chamber for 60 min. Finally, the tissue sections were immersed in 2 × SSC for 15 min to stop reaction. After washing, the tissue sections were counterstained with DAPI to stain nuclei. Localized green fluorescence of apoptotic tissue was detected by a laser scanning confocal microscope (PCM 200, Nikon).

### Flow Cytometric analysis

HEK293FT cells were collected by centrifugation at 200 × *g* for 5 min and resuspended in a binding buffer (10 mM HEPES, 140 mM NaCl, 2.5 mM CaCl_2_) at room temperature at a density of 1 × 10^6^ cell/ml. The cells (100 μl) were mixed with 5 μl of annexin-V-FITC (BD Biosciences, Shanghai, China) and 5 μl of PI in a culture tube and incubated at room temperature in the dark for 15 min. After addition of 400 μl binding buffer, the cells (~10,000 cells per assay) were then analyzed by using a dual-laser FACS VantageSE flow cytometer (Becton Dickinson, Mountain View, CA) within one hour period. The percentages of apoptotic cells at early stage for each sample were estimated.

### Statistical analysis

All experiments were carried out at least three times. ANOVA and Student’s *t*-test were performed to determine the significance of differences between groups, and a *p*-value of <0.05 was defined to be statistically significant.

## Additional Information

**How to cite this article**: Shi, J. *et al.* O-GlcNAcylation regulates ischemia-induced neuronal apoptosis through AKT signaling. *Sci. Rep.* **5**, 14500; 10.1038/srep14500 (2015).
